# Relation between *tetR* and *tetA* expression in tetracycline resistant Escherichia *coli*

**DOI:** 10.1186/s12866-016-0649-z

**Published:** 2016-03-12

**Authors:** Thea S. B. Møller, Martin Overgaard, Søren S. Nielsen, Valeria Bortolaia, Morten O. A Sommer, Luca Guardabassi, John E. Olsen

**Affiliations:** Department of Veterinary Disease Biology, Faculty of Health and Medical Sciences, University of Copenhagen, Frederiksberg, C Denmark; Department of Clinical Biochemistry and Pharmacology, Odense University Hospital, Odense, Denmark; Institute of Clinical Research, University of Southern Denmark, Odense, Denmark; Department of Large Animal Sciences, Faculty of Health and Medical Sciences, University of Copenhagen, Frederiksberg, C Denmark; Department of Systems Biology, Technical University of Denmark, Lyngby, Denmark; Novo Nordisk Foundation Centre for Biosustainability, Technical University of Denmark, Hørsholm, Denmark

**Keywords:** Antimicrobial resistance, TetA, TetR, Tetracycline, Tetracycline resistance

## Abstract

**Background:**

Tetracyclines are among the most used antibiotics in livestock worldwide. Resistance is widely disseminated in *Escherichia coli,* where it is generally mediated by tetracycline efflux pumps, such as TetA. Expression of tetracycline efflux pumps is tightly controlled by the repressor TetR, which has been shown to be tetracycline-responsive at sub-MIC tetracycline concentrations. The objective of this study was to investigate the effects of increasing tetracycline concentrations on the growth of TetA-producing *E. coli*, and to determine how expression of *tetA* and *tetR* related to each other in different growth phases in the presence of tetracycline.

**Results:**

A tetracycline resistant *E. coli* strain containing *tetA* and *tetR* on the chromosome was constructed and cultured in the presence of increasing concentrations of tetracycline. Expression of *tetR* and *tetA* was measured at four time points in different growth phases by quantitative real-time PCR. The TetA-producing *E. coli* exhibited prolonged lag phase with increasing concentrations of tetracycline, while expression of *tetA* and *tetR* increased and decreased, respectively, with increasing tetracycline concentration. The levels of *tetA* and *tetR* mRNA varied depending on growth phase, resulting in a gradual decrease of the *tetA*/*tetR* ratio from approximately 4 in the lag phase to approximately 2 in the stationary phase.

**Conclusion:**

This study shows that the expression of *tetR* and *tetA* is tetracycline concentration- and growth phase-dependent, contributing to improved understanding of the relationships between *E. coli* growth, tetracycline exposure and expression of tetracycline resistance.

**Electronic supplementary material:**

The online version of this article (doi:10.1186/s12866-016-0649-z) contains supplementary material, which is available to authorized users.

## Background

Tetracyclines exert their bacteriostatic activity by binding to the bacterial ribosome and thereby interfering with protein translation [1]. Over the years, the prevalence of tetracycline resistance has increased in bacteria isolated from both human patients [2, 3] and animals [4]. In a study performed by Tadesse *et al.* it was shown that the prevalence of tetracycline resistance in *Escherichia coli* isolates from humans increased by 0.45 % per year from 1950 to 2001 [5]. As a consequence of the spread of tetracycline resistance and the introduction of newer and more effective antimicrobial agents, the use of tetracylines in human medicine has gradually decreased. However, tetracyclines remain among the most used antibiotics in livestock production worldwide [6]. In the European Union, they account for 37 % of the total sales of antimicrobial agents for livestock [7] and a significant association has been observed at the country level between tetracycline consumption and occurrence of tetracycline resistance among *E. coli* isolated from livestock [8].

More than 40 genes encoding tetracycline resistance (*tet-*genes) have been characterized to date and they are divided into 11 classes, with a majority of classes (60 %) encoding for membrane-associated efflux proteins [9–11]. These efflux pumps selectively transport tetracycline from the cytosol to the periplasm in exchange of a proton, thereby limiting the access of tetracycline to the ribosomes in the cell [12]. They are proton motive force-dependent, single polypeptide, drug specific efflux pumps, which belong to the major facilitator superfamily [12, 13]. The flow of protons through the pump provides the required energy to pump the antibiotic to the periplasm [9, 14]. In *E. coli*, *tet-*resistance efflux pumps are among the best characterized transport systems [12]. Studies from selected parts of the United States of America list TetA as the second most frequent tetracycline resistance efflux pump in both human and animal isolates [15]. TetA is also the most common tetracycline efflux pump type found in clinical as well as commensal isolates of *E. coli* in animals from Denmark [4].

**Fig. 1:**
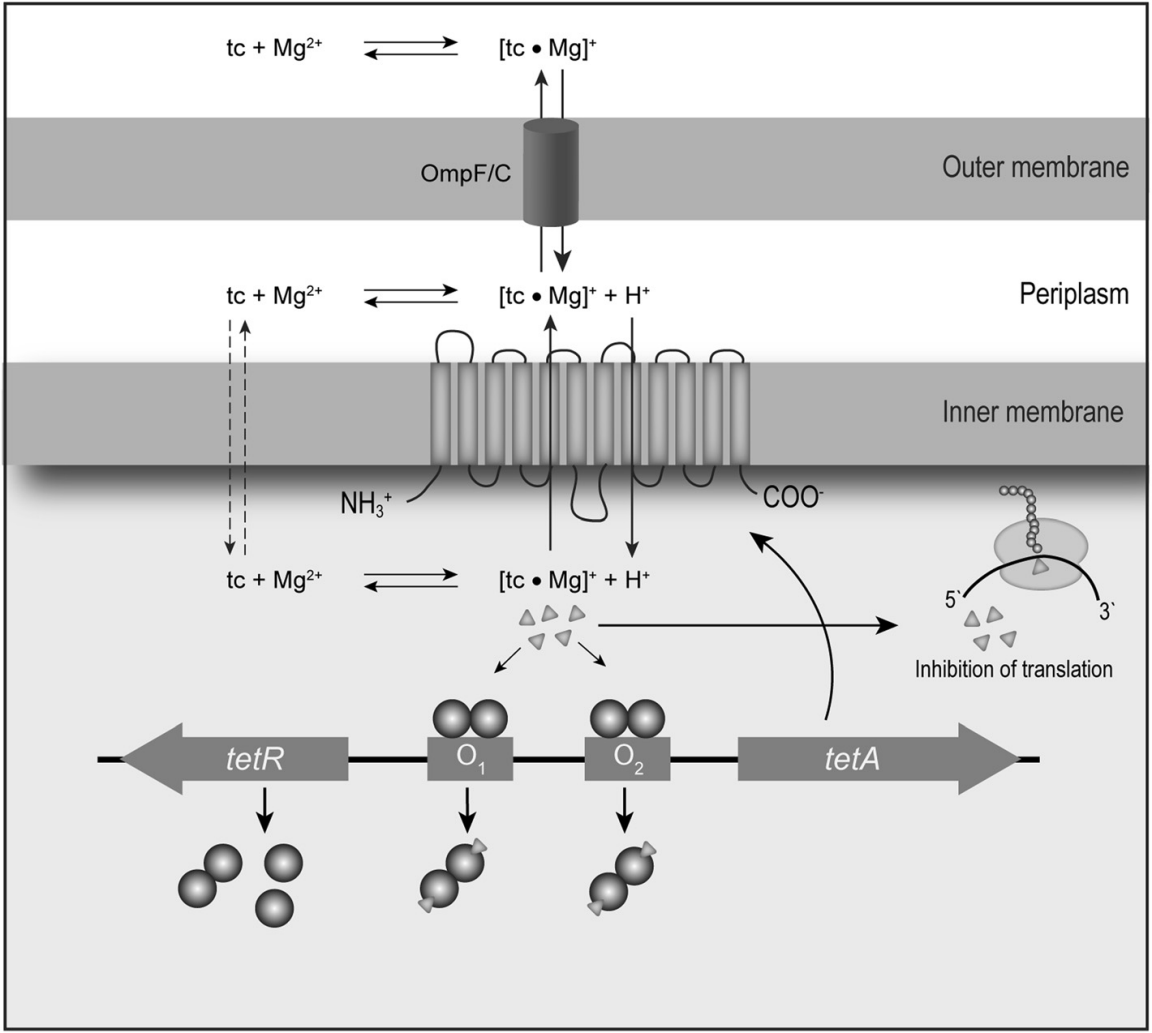
*TetR-tetA* regulation model. Genetic organisation and mechanism of regulation of the Tn*10*-encoded tetracycline resistance determinant as proposed by Hillen and Berens [24]. The upper part of the figure describes processes occurring at the cytoplasmic membrane, while the lower part shows a schematic drawing of the genetic organisation and induction of the *tetR* and *tetA* genes. Tetracycline (tc) is assumed to enter the outer membrane by porins [1, 46]. The [tc · Mg]^+^ complex is formed, however it can dissociate and tetracycline can enter the cell by diffusion across the cytoplasmic membrane in its neutral form (middle). In the cytoplasm the [tc · Mg]^+^ complex is formed again. The same complex, indicated by triangles, is the molecular inducer of the Tet repressor (circles), encoded by *tetR* (thick grey arrow, bottom). TetR forms a dimer and binds to the two tandem *tet* operators O_1_ and O_2_ located between *tetA* and *tetR* in the absence of tetracycline. The genes have divergent polarities and are transcribed from overlapping promoters. When tetracycline is present, the [tc · Mg]^+^ complex binds to the TetR repressor-operator complex and triggers a conformational change in TetR so that it can no longer bind to the *tet*-operators, enforcing rapid dissociation from the DNA, enabling transcription of *tetR* and *tetA* (bottom, center). The [tc · Mg]^+^ complex is the substrate of the proton-tetracycline antiporter (TetA pump) which is indicated by its 12-α-helix membrane spanning structure. Modified from [9, 24]

Expression of TetA efflux pump is controlled through a tetracycline-responsive repressor, TetR, which tightly regulates the *tetA* mRNA expression [16, 17]. Studies performed in the 1960-80s with sub-inhibitory (i.e. below the minimum inhibitory concentration, MIC) concentrations of tetracycline and derivatives thereof [18–23] led to a widely accepted model for *tetR* and tetracycline efflux pump gene regulation proposed by Hillen and Berens [24]. According to this model, tetracycline induces transcription of both the *tetR* and the tetracycline efflux pump gene by binding to TetR (Fig. 1) [24–26]. Repression by TetR must be tight, because constitutive expression of the efflux pump strongly reduces bacterial fitness [27]. It has never been determined how *tetR* and the tetracycline efflux pump gene expression varies over a range of tetracycline concentrations, and whether the model description of regulation at low tetracycline concentrations fits tetracycline resistant *E. coli* that are faced with fighting therapeutic concentrations of the antibiotic. The aim of the current study was to investigate the growth response of TetA-producing *E. coli* over a wide range of tetracycline concentrations and to determine how *tetA* and *tetR* expression were influenced by tetracycline concentrations and growth phase.

## Methods

### Bacterial strain construction

Bacteria and plasmids used in this study are listed in Table 1. The *tetA* gene and its repressor *tetR* isolated from an IncN plasmid obtained from *E. coli* of sow origin [28] were cloned into the pseudo gene *ybeM* [29] on the chromosome of *E. coli* K-12 MG1655, resulting in strain MG1655/TetA. The *tetA* and *tetR* genes were cloned into the chromosome of a laboratory *E. coli* strain to avoid problems related to the plasmid (e.g. other resistance genes encoded on the plasmid) and gene copy number. The Lambda Red recombination system was used to construct the strain, as described previously [30–32]. Sequences of oligonucleotides used for PCR verifications and Lambda Red mediated mutagenesis are listed in Table 2. Insertions were confirmed by PCR and sequencing using standard procedures. The strain was maintained in Difco™ Lysogeny broth (LB), Lennox (Becton, Dickinson and Company, Albertslund, Denmark) and on LB agar plates (Becton, Dickinson and Company, Albertslund, Denmark). The media were supplemented with tetracycline (10 μg/mL) (Sigma, Copenhagen, Denmark) when appropriate.

**Table 1:** Bacterial strains and plasmids used in this study

Strain or plasmid	Genotype	Reference or source
*E. coli* K-12 MG1655	Wild type	[47]
MG1655/TetA	*E. coli* MG1655 ∆YbeM::TetA (tet^r^)	This work
IncN/TetA	*tetR* and *tetA* containing plasmid	[28]
pKD46-gm	Vector for Lambda Red mediated mutagenesis: λ-red expression from arabinose-inducible promoter; temperature sensitive (gm^r^)	[30]
*E. coli* ATCC 25922	Reference strain	[48]

**Table 2:** Oligonucletide sequences for PCR based amplification and sequencing

Primer	Sequence	Application
TetA-ybeM	for: 5’-ATGCTGGTGGCACTTCAGGCAGGAAACATCGTCGCCCGTTCAATCGTCACCCTTTCTCGG -3’	Recombination
	rev: 5’-AGGCGGCAGGAAGTACCAGGATTTCAGCTCCCTGTCTATGTGCAACGGGAATTTGAAG-3’	
TetA/ybeM	for: 5’-GAAGCATTGCTGGCGCGCGATG-3’	Proof of insertion
	rev: 5’-TTCTCCAAAGCCCGCGACGCAG-	
TetA	for: 5’-AAGAATCCGCGCGTTCAATCG-3’	Sequencing
	rev: 5’-GCCCGGCACCGGCATAAT-3’	
TetR	for: 5’-TCGGATCCTCAATCGTCACCCTTT-3’	Sequencing
	for: 5’-CGCATCCATGCCGGCACG-3’	
NusG	for: 5’-GTCCGTTCGCAGACTTTAAC-3’	qPCR
	rev: 5’-GCTTTCTCAACCTGACTGAAG-3’	
GapA	for: 5’-ACTGACTGGTATGGCGTTCC-3’	qPCR
	rev: 5’-GTTGCAGCTTTTTCCAGACG-3’	
TetA-qPCR	for: 5’-CGGTCTTCTTCATCATGCAAC -3’	qPCR
	rev: 5’-GTCCCAGTGAAAGCGATCC-3’	
TetR-qPCR	for: 5’-CCGAATGCGTATGATTCTCC-3’	qPCR
	rev: 5’-CGCTTTACTGGCACTTCAGC-3’	

### Antimicrobial susceptibility testing

The broth microdilution method was used to determine the MIC of tetracycline following the CLSI guidelines [33]. The tetracycline concentrations tested ranged from 0 to 1024 μg/mL by two-fold dilution increase. In addition, MIC determination was performed using serial increases of 2 μg/mL of tetracycline in the range close to the observed MIC to obtain a value as close to the real MIC as possible. Furthermore, MIC experiments with spent media containing tetracycline (MG1655/TetA incubated in MH-2 with 8 μg/mL tetracycline for 15 hours at 37 °C, centrifuged and filter sterilised) and media with tetracycline (MH-2 with 8 μg/mL tetracycline incubated for 15 hours at 37 °C) were performed using the same method to test for drug degradation and destruction.

### Growth conditions

A BioScreen CTM was used to perform growth experiments for 24 hours at 37 °C in biological triplicates. A volume of 200 μL Müller-Hinton-II (MH-2) broth (Sigma, Copenhagen, Denmark) was inoculated with cells from blood agar plates (blood agar base (Oxoid, Roskilde, Denmark) supplemented with 5 % blood from cattle) to a final cell density of 10^6^ cfu/mL, using a Sensititre™ Nephelometer (Thermo Scientific™, Roskilde, Denmark) with a McFarland 0.5 standard (1–2 x 10^8^ cfu/mL). The cultures were supplemented with tetracycline (ranging from 0 to 128 μg/mL by two-fold dilutions). Optical density (OD, recorded with 600 nm filter) was measured every 5 minutes keeping bacterial cultures under continuous shaking. The specific maximum growth rate at different tetracycline concentrations was calculated. For expression studies, the strain was grown in 100 mL of MH-2 broth in 250 mL flasks at 37 °C and 225 rpm. The medium was supplemented with three different concentrations of tetracycline representing ^1^/_8_ MIC (3.5 μg/mL), ¼ MIC (7 μg/mL) and ½ MIC (14 μg/mL) of the strain and inoculated with a preculture grown for 2 hours at 37 °C and 225 rpm using the method described above. The tetracycline concentrations represent therapeutic concentrations, according to published pharmacokinetics data [34]. Although it has been shown that tetracycline in water solution is stable for 3 days at 37 °C [35], and approximately 5 % tetracycline is degraded after 2 days at 40 °C [36], we ensured that the late growth onset in our growth studies was not due to degradation of tetracycline by using an *E. coli* reference strain (ATCC 25922) with a MIC of tetracycline of 2 μg/mL [37]. *E. coli* ATCC 25922 was grown with the same tetracycline concentrations as used for the tetracycline resistant strain and MIC values were determined using spent media.

### RNA extraction

Samples for RNA extraction were collected at four different time points during *in vitro* growth; the lag phase (OD_600nm_ = 0.1-0.2), the logarithmic phase (OD_600nm_ = 0.5-0.6), the late logarithmic phase (OD_600nm_ = 1-1.3) and the stationary phase (OD_600nm_ = 3.3-4.6). Sampling, RNA extraction, DNase treatment and reverse transcription were performed as previously described [38].

### Quantitative real time polymerase chain reaction

Quantitative real time polymerase chain reaction (qPCR) was performed using a LightCycler 96 (Roche, Hvidovre, Denmark) as described previously [38]. Primers are listed in Table 2. The genes *gapA* and *nusG* were used as reference genes according to previous validation experiments [38]. Relative gene expression (fold change) was calculated compared to the lag phase sample of strain MG1655/TetA without antibiotics. Two independent biological replicates were performed using two technical replicates and the 2^-∆∆Ct^ method, corrected by different primer efficiencies and multiple reference genes was used [39].

### Statistical analysis

Statistical analysis was performed as previously described [38]. Briefly, the differences in the normalised qPCR measurements between the tetracycline concentrations within each growth phase were compared by differences in least square means using analysis of variance. The Mixed procedure in SAS version 9.3 (SAS Institute, Cary, USA) was used and differences in least square means estimates were evaluated with an approximate t-test using the LSmeans-function. Differences between tetracycline concentrations and growth phases were evaluated by F-tests, and a P value < 0.05 was deemed statistically significant. To correct for multiple comparisons of the differences in the least-squares means, the Benjamini–Hockberg ‘false discovery rate’ (FDR) was used [40].

## Results and Discussion

### Lag phase duration increases in the presence of tetracycline in a concentration-dependent manner

**Fig. 2:**
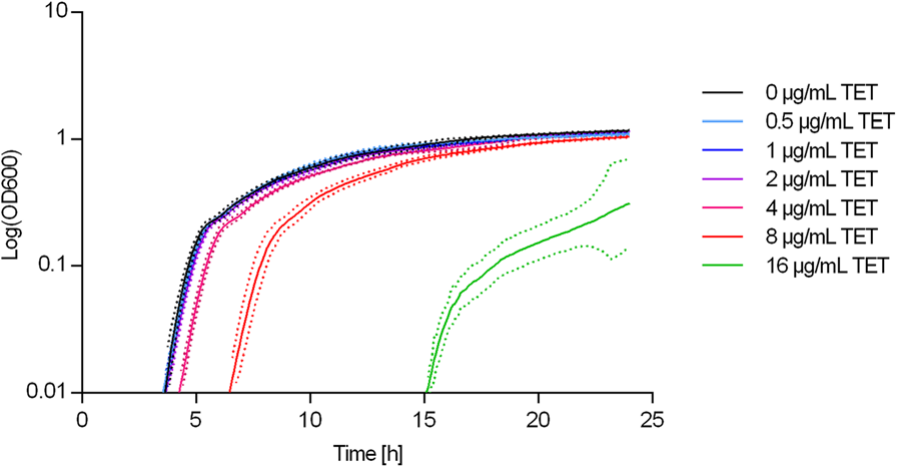
Growth curves of the TetA-producing *E. coli* strain at different concentrations of tetracycline. *E. coli* MG1655 containing *tetR* and *tetA* on the chromosome (MG1655/TetA). The strain was grown in MH-2 broth with different concentrations of tetracycline (TET) on a BioScreen CTM. No growth was observed for the three highest concentrations tested. Three independent replicates were performed; the data shown represents the mean and dots represent standard deviations

The MIC of tetracycline for MG1655/TetA was 28 ± 2 μg/mL. Using a definition of lag phase as the time necessary to reach an OD_600nm_ of 0.1, the lag phase length increased when the tetracycline concentration was above 2 μg/mL (Figure 2 and Additional file 1: Table S1). The lag phase length increased from 4.28 ± 0.10 hours at 4 μg/mL tetracycline to 18.17 ± 3.67 hours at 16 μg/mL tetracycline. In accordance with the MIC value, MG1655/TetA did not grow at tetracycline concentrations above 16 μg/mL (Fig. 2). The ATCC 25922 reference strain did not display growth at tetracycline concentrations other than 0 μg/mL and 0.5 μg/mL (data not shown). With 0.5 μg/mL of tetracycline, the control strain started to grow after 19 hours. No growth was observed above the MIC of the reference strain (2 μg/mL) [37]. Furthermore, MIC experiments with spent media containing tetracycline and media incubated with tetracycline were performed, and the reference strain showed the same MIC towards tetracycline as using fresh tetracycline. These experiments show that the tetracycline did not degrade during the growth experiment, and that the prolonged lag phase observed in the experiment was not attributable to drug degradation. A possible explanation to the long lag phases observed in the presence of tetracycline is that the levels of tetracycline need to be below a certain threshold in the cytosol before growth can initiate. Based on the results of this experiment, the time to reach this threshold level appears to be tetracycline concentration-dependent. It is speculated that tetracycline concentrations up to a certain level could be dealt with by increasing the numbers of TetA pumps in the membrane as the lag phase was unchanged. Then a maximum level of pumps was reached, and the lag phase increased as there presumably were too few TetA pumps to deal with the high tetracycline concentration.

The maximum specific growth rate of the strain decreased with increasing tetracycline concentrations (Additional file 1: Table S1), showing that increased tetracycline concentrations resulted in slower growth. However, this could not account for the increased lag phase. Since the concentrations we have investigated are within the range of those achieved during therapy [34], these observations indicate that even in tetracycline-resistant strains growth is delayed during therapy. A recent model study of growth kinetics of tetracycline-resistant strains during therapy in the gut of post weaning pigs showed that resistant strains having a 10 % reduction in the growth rate still could colonize and remain in the gut in a stable manner despite this disadvantage [41].

### Expression of tetR mRNA is tetracycline concentration- and growth phase-dependent

**Fig. 3:**
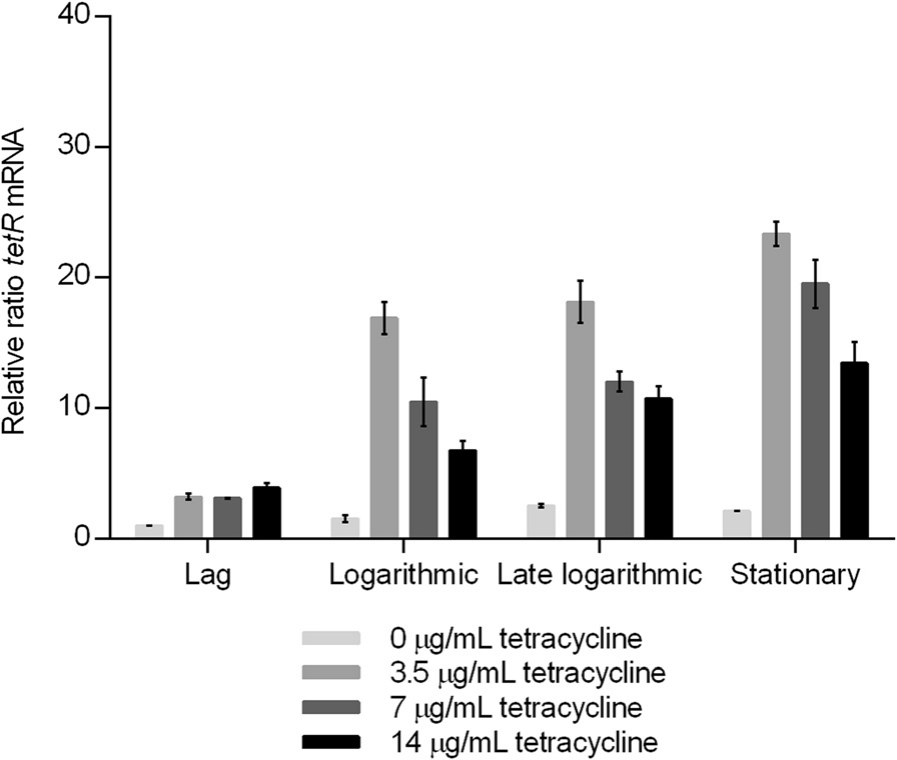
Relative changes in *tetR* mRNA levels. *tetR* mRNA profile of *E. coli* MG1655 containing *tetA* and *tetR* on the chromosome (MG1655/TetA). Growth was performed in MH-2 broth without and with tetracycline at different concentrations. Two independent replicates including two technical replicates each were performed; the data shown represents the mean. The data have been normalised to two validated reference genes, *gapA* and *nusG*, and are relative to the *tetR* mRNA level in the lag phase with no antibiotics. The error bars represent standard deviations

The mRNA levels of *tetR* were significantly higher in samples with the presence of tetracycline compared to the absence of tetracycline (Fig. 3). A significant 3 to 25 fold increase was observed in the mRNA when the strain was exposed to 3.5 μg/mL of tetracycline in the different growth phases (adjusted P-values: 0.019 in lag phase, 0.002 in log phase, 0.005 in late log phase and 0.001 in stationary phase). Significantly lower *tetR* mRNA levels occurred when the strain was exposed to 7 μg/mL or 14 μg/mL of tetracycline, even though they were significantly higher than the levels observed in absence of tetracycline (Fig. 3). The decrease in expression with increasing tetracycline concentrations was observed in all growth phases, except in the lag phase. The decrease in the mRNA level was significant between the samples with 3.5 and 14 μg/mL tetracycline in the logarithmic and late logarithmic phase (adjusted P-values: 0.023 and 0.029, respectively). All statistically significant P-values of the observed differences are listed in Additional file 1: Table S2. Overall, expression of *tetR* was shown to be tetracycline concentration-dependent. The observed fluctuations of *tetR* mRNA levels in cells exposed to different concentrations of tetracycline represent valuable knowledge when considering new treatment strategies for tetracycline resistant *E. coli* strains. When treating infections with tetracycline-resistant bacteria, it may not always be optimal to increase the tetracycline concentration as the *tetR* mRNA level will decrease, and thereby affect the *tetA* mRNA level (see below).

Expression of *tetR* at a specific tetracycline concentration varied depending on growth phase (Fig. 3). The level of *tetR* mRNA in MG1655/TetA increased from lag to stationary phase at all concentrations tested. Statistically significant increases in the *tetR* mRNA level were observed from logarithmic to stationary phase when the strain was exposed to 7 μg/mL or 14 μg/mL of tetracycline (adjusted P-values: 0.026 and 0.025, respectively, see Additional file 1: Table S2).

### Expression of tetA mRNA is also tetracycline- and growth phase-dependent

**Fig. 4:**
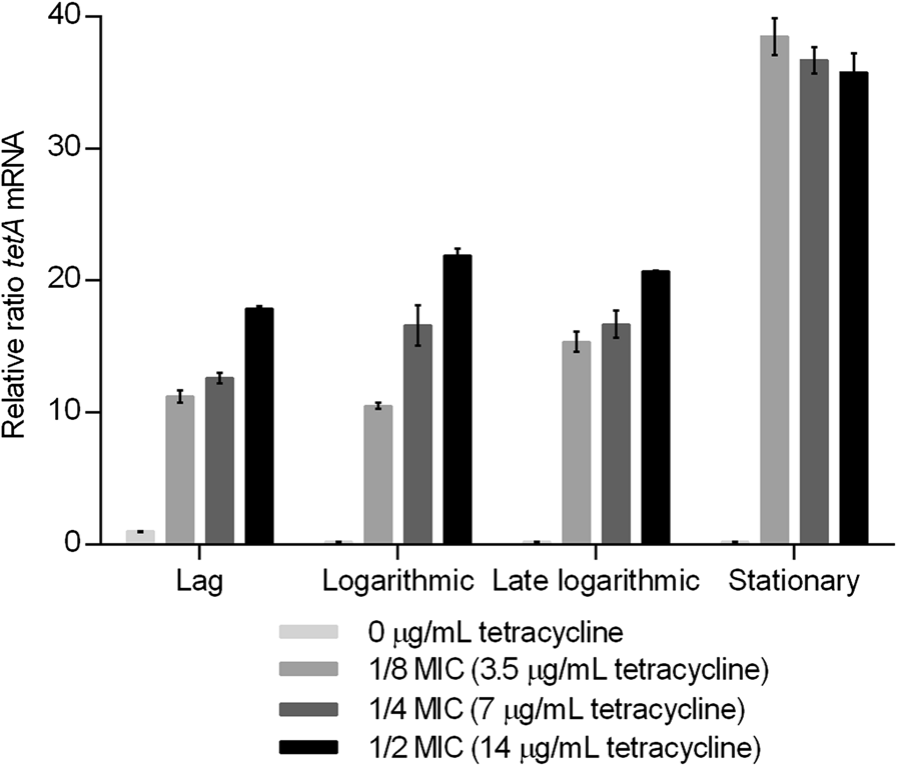
Relative changes in *tetA* mRNA levels. *tetA* mRNA profile of *E. coli* MG1655 containing *tetA* and *tetR* on the chromosome (MG1655/TetA). The strain was grown in MH-2 broth without and with tetracycline at different concentrations. Two independent replicates including two technical replicates each were performed; the data shown represents the mean. The data have been normalised to two validated reference genes, *gapA* and *nusG,* and are relative to the *tetA* mRNA level in the lag phase with no antibiotics. The error bars represent standard deviations

Expression of *tetA* increased significantly when tetracycline was present (Fig. 4), which is in accordance with the previously described *tetR-tetA* regulation model [24]. Significant *tetA* mRNA level increments were observed in the presence of tetracycline regardless of growth phase (Additional file 1: Table S3). The *tetA* mRNA level showed a tendency to increase with increasing concentrations of tetracycline in the lag, logarithmic and late logarithmic growth phases. Significant P-values for tetracycline concentration-dependency are listed in Additional file 1: Table S3. These results confirm that *tetA* expression is dependent on the presence of tetracycline and influenced by the drug concentration. Despite several attempts by us to determine the TetA protein levels in the strain by selected-reaction-monitoring mass spectrometry (SRM-MS), it failed because the TetA protein could not be specifically detected (methods and data not shown). Similarly, western blot experiments could not be performed as TetA could not be overproduced in order to get enough purified protein to produce specific antibodies. When TetA overproduction was attempted by induction of a His-tagged or glutathione S-transferase-tagged *tetA* with IPTG, to facilitate purification, bacterial cell viability was lost (data not shown), which is in accordance with previous observations by Eckert *et al.*^247^ Therefore it is currently unknown whether high *tetA* mRNA levels lead to proportionally high levels of TetA pumps. Assuming a positive correlation, the results indicate that exposing tetracycline resistant *E. coli* to high tetracycline concentrations will result in an increased *tetA* mRNA level and therefore a higher number of TetA pumps.

The *tetA* mRNA levels also varied according to growth phase with different patterns depending on the presence of tetracycline (Fig. 4). A decrease in *tetA* mRNA was observed from lag to stationary phase in the absence of tetracycline. In the presence of fixed concentrations of tetracycline, the level of *tetA* mRNA increased from lag to logarithmic phase (except in the logarithmic phase at 3.5 μg/mL tetracycline) and stabilised in the late logarithmic phase. In the stationary phase, the *tetA* mRNA level was approximately twice of that observed in the late logarithmic growth phase regardless of the drug concentration to which the strain was exposed. Significant P-values for growth phase dependency can be found in Additional file 1: Table S3.

### tetA/tetR mRNA ratio

**Fig. 5:**
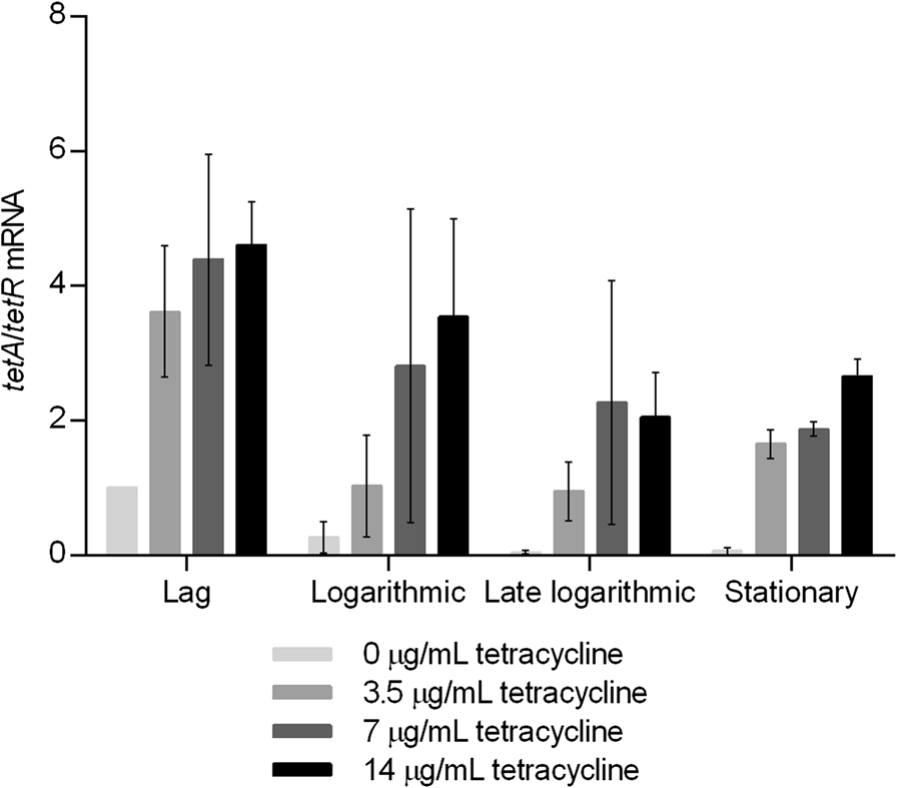
*tetA*/*tetR* mRNA ratio. tetA/tetR mRNA ratios were calculated for each biological replicate. The error bars represent standard deviations

To observe more clearly how the mRNA level of *tetA* related to *tetR* expression, the *tetA*/*tetR* ratio was calculated (Fig. 5). In all growth phases except the lag phase, the *tetA/tetR* mRNA ratio was low in absence of tetracycline, indicating low mRNA level of *tetA* compared to *tetR*. This corroborates the model assumption that TetR needs to be produced to block the expression of *tetA.* When the strain was exposed to any of the tetracycline concentrations used, which are above previously tested concentrations, the *tetA*/*tetR* ratio increased markedly, showing that much more *tetA* mRNA was present compared to *tetR* mRNA, and this was especially observed in the lag phase, where the *tetA*/*tetR* ratio was in the range of 3.5-4.5 fold. Furthermore, the *tetA*/*tetR* ratio was tetracycline concentration-dependent with higher *tetA/tetR* mRNA ratios at higher drug concentrations in all growth phases.

It is known that the TetR repressor protein has a 2-fold higher affinity for the *tetA* proximal operator O_2_ site compared to the *tetR* proximal operator O_1_ site [42]. This can explain why much lower *tetA/tetR* mRNA ratios were observed in the absence of tetracycline induction. In the model, tetracycline binds to Mg^2+^, and these complexes bind to TetR dimers and cause a conformational change that makes the TetR protein unable to bind to the operator sites O_1_ and O_2_. This turns on the expression of both *tetA* and *tetR* in a tetracycline-dependent manner. In a previous study the *tetA* promoter was found to be about four times as strong as the two overlapping *tetR* promoters together [43]. Another study performed by Daniels *et al.* showed that the *tetA* promoter was 7–11 fold more active than the *tetR* promoters combined [44]. A stronger *tetA* promoter corresponds well with the rise in *tetA/tetR* mRNA ratios in our studies with increasing concentrations of tetracycline in the lag phase.

A tetracycline concentration-dependent change in *tetA/tetR* mRNA ratio was found between growth phases with a decrease from onset of growth to maximum growth rate. We speculate that once growth is in the exponential phase, each cell contains the maximum level of TetA pumps in the membrane. This facilitates that enough tetracycline is pumped out to reach a critical concentration below which TetR is again free to bind the operator sites. TetR preferably binds to O_2_, which results in blocking the transcription of *tetA* but not of *tetR* [45]. This explain the increase in *tetR* mRNA from lag to logarithmic phase, the constant *tetA* mRNA level in lag, logarithmic and late logarithmic phase (Figs. 3 and 4, respectively), and the lowered *tetA/tetR* mRNA ratios in the logarithmic, late logarithmic and stationary growth phases compared to the lag phase (Fig. 5). The highest *tetA* and *tetR* mRNA levels were observed at stationary phase in the presence of tetracycline. However, interpretation of transcriptional regulation from stationary growth phase cultures should be done with caution due to the cell complexity with a mixture of cells in different physiological phases.

## Conclusions

This study shows that a tetracycline resistant TetA-producing *E. coli* exhibited prolonged lag phase with increasing concentrations of tetracycline. This suggests that even tetracycline-resistant strains are growth-detained in the presence of tetracycline, likely due to the time needed to express the resistance genes and export tetracycline out of the cell. The expression of *tetA* and *tetR* increased and decreased, respectively, with increasing tetracycline concentration. Furthermore, expression of *tetA* and *tetR* was shown to be growth phase-dependent, and specifically increased from lag to stationary phase in the presence of tetracycline. However, such a growth-dependent increase was not proportional for the two genes, resulting in a gradual decrease of the *tetA*/*tetR* mRNA ratio from lag to stationary phases. The basic observations behind the *tetR*-*tetA* regulation model [24], the affinity studies and promoter strength studies [42, 44] previously used to explain regulation of TetA production at low concentrations of tetracycline also seem to be applicable to the data obtained in the current study, which shows that the tetracycline regulation model also applies at therapeutic tetracycline concentrations. Altogether these results contribute to improve understanding of the relationships between *E. coli* growth, exposure to tetracycline and expression of tetracycline resistance.

## Additional file

Additional file 1: Table S1. Lag phase length and specific maximum growth rate. Length of lag phase and the specific maximum growth rate of MG1655/TetA at different tetracycline concentrations. The length of the lag phase was defined as the time necessary for the culture to reach an OD600nm of 0.01. **Table S2.** Significant P-values for differences in *tetR* expression levels measured at different tetracycline concentrations and growth phases. Adjusted significant P-values (P < 0.05) for tetracycline concentration-dependent and growth phase-dependent differences of *tetR* mRNA levels determined by qPCR for MG1655/TetA. **Table S3.** Significant P-values for differences in *tetA* expression levels measured at different tetracycline concentrations and growth phases. Adjusted significant P-values (P < 0.05) for tetracycline concentration-dependent and growth phase-dependent differences of *tetA* mRNA levels determined by qPCR for MG1655/TetA. (DOC 90 kb)
