# Supplementary material for: Relation between tetR and tetA expression in tetracycline resistant Escherichia coli
Source: BMC Microbiol. 2016 Mar 12;16:39. doi: 10.1186/s12866-016-0649-z (PMC4788846; doi:10.1186/s12866-016-0649-z)
Supplement: Additional file 1: Table S1. — Lag phase length and specific maximum growth rate. Length of lag phase and the specific maximum growth rate of MG1655/TetA at different tetracycline concentrations. The length of the lag phase was defined as the time necessary for the culture to reach an OD600nm of 0.01. Table S2. Significant P-values for differences in tetR expression levels measured at different tetracycline concentrations and growth phases. Adjusted significant P-values (P < 0.05) for tetracycline concentration-dependent and growth phase-dependent differences of tetR mRNA levels determined by qPCR for MG1655/TetA. Table S3. Significant P-values for differences in tetA expression levels measured at different tetracycline concentrations and growth phases. Adjusted significant P-values (P < 0.05) for tetracycline concentration-dependent and growth phase-dependent differences of tetA mRNA levels determined by qPCR for MG1655/TetA. (DOC 90 kb) [file 12866_2016_649_MOESM1_ESM.doc]

***S1 Table. Lag phase length and specific maximum growth rate.*** *Length of lag phase and the specific maximum growth rate of MG1655/TetA at different tetracycline concentrations. The length of the lag phase was defined as the time necessary for the culture to reach an OD600nm of 0.01.*

| **MG1655/TetA** | | |
| --- | --- | --- |
| Antibiotic concentration [μg/mL] | Length of lag phase [h] | Max growth rate |
| 0 | 3.64 ± 0.21 | 0.95 ± 0.12 |
| 0.5 | 3.58 ± 0.07 | 0.86 ± 0.01 |
| 1 | 3.64 ± 0.04 | 0.86 ± 0.01 |
| 2 | 3.73 ± 0.08 | 0.83 ± 0.04 |
| 4 | 4.28 ± 0.10 | 0.87 ± 0.02 |
| 8 | 6.48 ± 0.19 | 0.78 ± 0.11 |
| 16 | 18.17 ± 3.67 | 0.75 ± 0.02 |

**S2 Table**. **Significant P-values for differences in *tetR* expression levels measured at different tetracycline concentrations and growth phases**. Adjusted significant P-values (P < 0.05) for tetracycline concentration-dependent and growth phase-dependent differences of *tetR* mRNA levels determined by qPCR for MG1655/TetA.

| ***tetR*** | | | | | | | |  | |
| --- | --- | --- | --- | --- | --- | --- | --- | --- | --- |
| **Tetracycline concentration-dependent 0000000 Growth phase-dependent000000** | | | | | | | |  | |
| **Phase** | **Conc.** | **Conc.** | **Adj. P-value** | **Conc.** | **Phase** | **Phase** | **Adj. P-value** | |  |
| lag | 0 | 3.5 | 0.019 | 0 | lag | late log | 0.022 | |  |
| lag | 0 | 7 | 0.020 | 0 | lag | st | 0.024 | |  |
| lag | 0 | 14 | 0.015 | 3.5 | lag | log | 0.010 | |  |
| log | 0 | 3.5 | 0.002 | 3.5 | lag | late log | 0.009 | |  |
| log | 0 | 7 | 0.006 | 3.5 | lag | st | 0.004 | |  |
| log | 0 | 14 | 0.013 | 7 | lag | log | 0.018 | |  |
| log | 3.5 | 14 | 0.023 | 7 | lag | late log | 0.016 | |  |
| late log | 0 | 3.5 | 0.005 | 7 | lag | st | 0.008 | |  |
| late log | 0 | 7 | 0.011 | 7 | log | st | 0.026 | |  |
| late log | 0 | 14 | 0.014 | 14 | lag | log | 0.028 | |  |
| late log | 3.5 | 14 | 0.029 | 14 | lag | late log | 0.021 | |  |
| st | 0 | 3.5 | 0.001 | 14 | lag | st | 0.017 | |  |
| st | 0 | 7 | 0.003 | 14 | log | st | 0.025 | |  |
| st | 0 | 14 | 0.007 |  |  |  |  | |  |

Lag: lag phase, log: logarithmic phase, late log: late logarithmic phase, st: stationary phase and

Conc.: concentration (μg/mL). Adjusted (Adj.) P-values are corrected for multiple comparisons by

Benjamini-Hockberg “false discovery rate” (FDR).

**S3 Table**. **Significant P-values for differences in *tetA* expression levels measured at different tetracycline concentrations and growth phases**. Adjusted significant P-values (P < 0.05) for tetracycline concentration-dependent and growth phase-dependent differences of *tetA* mRNA levels determined by qPCR for MG1655/TetA

| ***tetA*** | | | | | | | |  | |
| --- | --- | --- | --- | --- | --- | --- | --- | --- | --- |
| **Tetracycline concentration-dependent 0000000 Growth phase-dependent000000** | | | | | | | |  | |
| **Phase** | **Conc.** | **Conc.** | **Adj. P-value** | **Conc.** | **Phase** | **Phase** | **Adj. P-value** | |  |
| lag | 0 | 3.5 | 0.013 | 0 | lag | log | 0.016 | |  |
| lag | 0 | 7 | 0.011 | 0 | lag | late log | 0.013 | |  |
| lag | 0 | 14 | 0.010 | 0 | lag | st | 0.014 | |  |
| log | 0 | 3.5 | 0.009 | 3.5 | lag | st | 0.017 | |  |
| log | 0 | 7 | 0.008 | 3.5 | log | st | 0.018 | |  |
| log | 0 | 14 | 0.007 | 3.5 | Late log | st | 0.020 | |  |
| late log | 0 | 3.5 | 0.006 | 7 | lag | st | 0.019 | |  |
| late log | 0 | 7 | 0.005 | 7 | log | st | 0.021 | |  |
| late log | 0 | 14 | 0.004 | 7 | late log | st | 0.022 | |  |
| st | 0 | 3.5 | 0.001 |  |  |  |  | |  |
| st | 0 | 7 | 0.002 |  |  |  |  | |  |
| st | 0 | 14 | 0.003 |  |  |  |  | |  |

Lag: lag phase, log: logarithmic phase, late log: late logarithmic phase, st: stationary phase and

Conc.: concentration (μg/mL). Adjusted (Adj.) P-values are corrected for multiple comparisons by

Benjamini-Hockberg “false discovery rate” (FDR).
